# Survival analysis: coping with non proportional hazards in randomized trials

**DOI:** 10.1186/1745-6215-12-S1-A136

**Published:** 2011-12-13

**Authors:** Patrick Royston, Mahesh KB Parmar

**Affiliations:** 1 Hub for Trials Methodology Research, MRC Clinical Trials Unit, London, UK

## 

Almost all trials with a censored time-to-event outcome are designed, powered and analysed with a target hazard ratio for comparing experimental and control treatments in mind. Differences in survival experience between trial arms are tested with a logrank test and usually illustrated using a Kaplan-Meier plot. We describe this as an analysis in the *probability domain*. The focus is often on estimating survival probabilities at particular times after randomization.

In the absence of censoring, we would almost certainly prefer to work in the *time domain*. We would analyse times to event directly, summarizing results in terms of means, SDs and confidence intervals for differences. We would illustrate the different survival experiences using comparative scatter plots of times to event, histograms and the like.

The probability domain paradigm works well in many cases, but the hazard ratio, as a meaningful summary measure, requires one crucial assumption: proportional hazards of the treatment effect. In some recent high-profile trial reports, the PH assumption has clearly been invalid. We consider and recommend a general analysis strategy, relevant to the time domain, that does not assume PH. It based on the idea of the restricted mean survival time (RMST). We discuss the motivation, definition and interpretation of RMST and suggest how it can be woven into a strategy for time to event trials in which non-PH is anticipated or discovered.

